# The gene encoding the ketogenic enzyme HMGCS2 displays a unique expression during gonad development in mice

**DOI:** 10.1371/journal.pone.0227411

**Published:** 2020-01-07

**Authors:** Stefan Bagheri-Fam, Huijun Chen, Sean Wilson, Katie Ayers, James Hughes, Frederique Sloan-Bena, Pierre Calvel, Gorjana Robevska, Beatriz Puisac, Kamila Kusz-Zamelczyk, Stefania Gimelli, Anna Spik, Jadwiga Jaruzelska, Alina Warenik-Szymankiewicz, Sultana Faradz, Serge Nef, Juan Pié, Paul Thomas, Andrew Sinclair, Dagmar Wilhelm

**Affiliations:** 1 Department of Anatomy & Neuroscience, The University of Melbourne, Melbourne, Australia; 2 Institute for Molecular Bioscience, The University of Queensland, Brisbane, Australia; 3 Murdoch Children’s Research Institute, Melbourne, Australia; 4 Department of Paediatrics, The University of Melbourne, Melbourne, Australia; 5 School of Biological Sciences, University of Adelaide, Adelaide, Australia; 6 Service of Genetic Medicine, University Geneva Hospitals, Geneva, Switzerland; 7 Department of Genetics, Medicine & Development, University of Geneva, Geneva, Switzerland; 8 Unit of Clinical Genetics and Functional Genomics, Department of Pharmacology-Physiology, School of Medicine, University of Zaragoza, CIBERER-GCV02 and ISS-Aragon, Zaragoza, Spain; 9 Institute of Human Genetics, Polish Academy of Sciences, Poznań, Poland; 10 Department of Gynecological Endocrinology Poznan University of Medical Sciences, Poznan, Poland; 11 Center for Biomedical Research Faculty of Medicine Diponegoro University (FMDU), Semarang, Indonesia; University of Hyderabad, INDIA

## Abstract

Disorders/differences of sex development (DSD) cause profound psychological and reproductive consequences for the affected individuals, however, most are still unexplained at the molecular level. Here, we present a novel gene, 3-hydroxy-3-methylglutaryl coenzyme A synthase 2 (*HMGCS2*), encoding a metabolic enzyme in the liver important for energy production from fatty acids, that shows an unusual expression pattern in developing fetal mouse gonads. Shortly after gonadal sex determination it is up-regulated in the developing testes following a very similar spatial and temporal pattern as the male-determining gene *Sry* in Sertoli cells before switching to ovarian enriched expression. To test if *Hmgcs2* is important for gonad development in mammals, we pursued two lines of investigations. Firstly, we generated *Hmgcs2*-null mice using CRISPR/Cas9 and found that these mice had gonads that developed normally even on a sensitized background. Secondly, we screened 46,XY DSD patients with gonadal dysgenesis and identified two unrelated patients with a deletion and a deleterious missense variant in *HMGCS2* respectively. However, both variants were heterozygous, suggesting that *HMGCS2* might not be the causative gene. Analysis of a larger number of patients in the future might shed more light into the possible association of HMGCS2 with human gonadal development.

## Introduction

Disorders or differences of sex development (DSD) are defined as congenital conditions in which the development of chromosomal, gonadal, or anatomical sex is atypical [1–3]. One such condition, 46,XY gonadal dysgenesis (46,XY GD), is caused by partial or complete disruption of testis development. 46,XY GD patients show a wide spectrum of phenotypes such as hypospadias, ambiguous genitalia, undescended or atrophic testes, and complete male-to-female sex reversal. In addition, 46,XY GD often results in infertility and an increased risk of gonadal cancer. Thus, this condition can have profound psychological and medical consequences for the individual. The first causative variant in 46,XY GD was identified in the testis-determining gene *SRY* located on the Y chromosome [4, 5]. Since then, our understanding of the molecular and cellular processes of testis determination and differentiation has significantly advanced. However, despite this as many as 60% of 46,XY GD cases are still unexplained at the molecular level [3, 6, 7].

In mammals, testes in an XY and ovaries in an XX individual develop from a common precursor, the genital ridges. In mouse, at around 11.5 days *post coitum* (dpc), transient expression of SRY in pre-Sertoli cells leads to the up-regulation of the related transcription factor SOX9, which drives the differentiation of the genital ridges into testes [8–13]. A key step during this process is the differentiation of Sertoli cells, which requires a high-glucose metabolism [14, 15]. Sertoli cells surround germ cells to form the testis cords. In the interstitium, between testis cords, Leydig cells differentiate to produce testosterone which is ultimately responsible for the development of the male phenotype [13]. If the male-determining genetic program is disrupted, the female genetic program, marked by the expression of *Wnt4*, *Rspo1*, and *Foxl2*, is induced and the genital ridge will differentiate into an ovary [13, 16–21].

The autosomal *HMGCS2* gene encodes mitochondrial 3-hydroxy-3-methylglutaryl coenzyme A synthase 2, one of the major control points of ketogenesis in the liver [22]. When blood glucose levels are low, such as during starvation and sustained exercise, *HMGCS2* expression is up-regulated in the liver and ketogenesis is induced during which acetyl-CoA, derived from fatty acid ß-oxidation, is converted into ketone bodies such as ß-hydroxybutyrate (ßHB) [22, 23]. These ketone bodies are then transported from the liver to other tissues where they can be re-converted to acetyl-CoA for energy production. In humans, homozygous or compound heterozygous variants in *HMGCS2* lead to HMGCS2 deficiency disorder (OMIM: 605911), a very rare, autosomal recessive metabolic disorder [24–27]. Patients are usually asymptomatic and only present with symptoms such as vomiting, hypoketotic hypoglycemia, or coma after infections or prolonged fasting [28].

There is emerging evidence that expression of HMGCS2 and ketogenesis is not restricted to the liver but is also evident in other tissues such as in the eye, the intestine, and adult gonads [29–31]. In retinal pigment epithelial cells, HMGCS2 produces ßHB, which is used as a metabolite by retinal cells [29]. Apart from providing energy from fatty acids, HMGCS2 is also involved in gene regulation. In the intestine, ßHB produced by HMGCS2 inhibits histone deacetylases, known inhibitors of gene expression [32], to induce the expression of differentiation markers underlying intestinal cell differentiation [30]. HMGCS2 expression was also discovered in steroidogenic cells of adult rat testes and ovaries, Leydig and theca cells respectively [31]. It was speculated that HMGCS2 could be involved in androgen production in these tissues [31]. In contrast, a role for HMGCS2 in fetal gonad development has not been described to date.

## Material & methods

### Ethical considerations

Protocols and use of animals were approved by the Animal Welfare Unit of the University of Queensland (approval # IMB/131/09/ARC) and the Anatomy & Neuroscience Animal Ethics Committee of the University of Melbourne (approval # 1513724 and # 1613957). All experiments were performed in accordance with relevant guidelines and regulations. All clinical investigations have been performed according to the Declaration of Helsinki principles. The first part of the study was approved by the Bioethics Committee at Poznan University of Medical Sciences (authorization number 817/13) and the Geneva Ethical Committee (CCER, authorization number 14–121). All participants in the massive parallel sequencing approach provided written informed consent as part of The Royal Children’s Hospital Ethics committee approved study (HREC22073) or their local institution (medical ethics committee of Dr Kariadi Hospital/FMDU, Semarang).

### Mouse strains

For gene and protein expression studies wildtype embryos were collected from timed matings of the outbred CD1 mouse strain, with noon of the day on which the mating plug was observed designated 0.5 days *post coitum* (dpc). For more accurate staging of embryos up to 12.5dpc, the tail somite (ts) stage was determined by counting the number of somites posterior to the hind limb [33]. Using this method, 10.5dpc corresponds to 8ts, 11.5dpc to 18ts, and 12.5dpc to 30ts. Genetic sex was determined by PCR as described previously [34].

*Hmgcs2*-null deletion mice were generated using CRISPR/CAS9 at the University of Adelaide. A high scoring CRISPR guide RNA (gRNA) targeting the *Hmgcs2* coding sequence (*UACAAUCCCUCCUGCUCCCCUGG*) was identified using the MIT CRISPR design tool (https://zlab.bio/guide-design-resources) and animals were generated as previously described [35]. Heterozygous *Hmgcs2*^-/+^ mice on a C57BL/6 background were intercrossed to generate control and homozygous *Hmgcs2*^-/-^ embryos. Genotyping analysis for the *Hmgcs2* locus (**S1 Table**) was performed using genomic DNA isolated from tail tissue.

*Hmgcs2*-null mice lacking one copy of the *Fgfr2c* gene were generated by crossing heterozygous *Fgfr2c*^-/+^ mice [36] on a C57BL/6 background, which contain a translational stop codon in *Fgfr2* exon 9, with heterozygous C57BL/6 *Hmgcs2*^-/+^ mice. Resultant double heterozygous *Fgfr2c*^-/+^;*Hmgcs2*^-/+^ mice were backcrossed with heterozygous *Hmgcs2*^-/+^ to generate control and XY *Fgfr2c*^−/+^;*Hmgcs2*^−/−^ embryos at 13.5 dpc and 15.5 dpc. Genotyping analysis for the *Fgfr2c* locus [36] and the *Hmgcs2* locus (S1 Table) was performed using genomic DNA isolated from tail tissue.

### Section *in situ* hybridization (ISH) and immunohistochemistry (IHC)

The ISH probe for *Hmgcs2* (NP_032282) was cloned by RT-PCR from RNA prepared from whole mouse embryos at 13.5 dpc. Primers used to generate the *Hmgcs2*-specific ISH probes are listed in **S1 Table**. Mouse embryos were fixed in 4% paraformaldehyde (PFA) in PBS (137 mM NaCl, 10 mM phosphate, 2.7 mM KCl, pH of 7.4) at 4°C, embedded in paraffin, and section ISH carried out as described previously [37]. Simultaneous detection of RNA and protein in tissue sections was carried out by section ISH as described above followed by immunohistochemistry (IHC) as described previously [38]. Images were taken with an Olympus BX-51 microscope.

### Immunofluorescence

Mouse embryos were fixed in 4% PFA in PBS at 4°C, embedded in paraffin, sectioned at 5μm, and immunofluorescence performed as described previously [39]. Primary antibodies used for this study were anti-HMGCS2 rabbit monoclonal (1:50; ab137043, Abcam), anti-SOX9 sheep polyclonal (1:100; [40]), anti-MVH goat polyclonal (1:200; AF2030, R&D systems), anti-AMH goat polyclonal (1:200; sc6886, Santa Cruz), anti-AMH goat polyclonal (1:50; AF1446 R&D systems), anti-SYCP3 mouse monoclonal (1:100; ab97672, Abcam), anti-FOXL2 rabbit polyclonal (1:300; [41]) and anti-CYP11A1 rabbit polyclonal [42]. Secondary antibodies used were donkey anti-rabbit Alexa 488, donkey anti-rabbit Alexa 568, donkey anti-goat Alexa 488, donkey anti-goat Alexa 546, donkey anti-mouse Alexa 488, and donkey anti-sheep Alexa 647 obtained from Invitrogen and used at 1:300. Images were taken with a Zeiss LSM 510 Meta confocal microscope at the Australian Cancer Research Foundation Dynamic Imaging Centre for Cancer Biology, University of Queensland and with a Zeiss LSM800 confocal microscope at the Biological Optical Microscopy Platform (BOMP) at the Department of Anatomy and Neuroscience, The University of Melbourne.

### Hematoxylin & eosin (H&E) staining

Embryos were harvested from at 13.5 and 15.5dpc, fixed in 4% PFA overnight and then embedded in paraffin. Paraffin blocks were sectioned at 5 μm and sections stained with hematoxylin and eosin (H&E) for histological analysis.

### Quantitative real-time (RT-qPCR) and droplet digital (RT-ddPCR) RT-PCR

RT-qPCR using SYBR green (Invitrogen) [43, 44] and RT-ddPCR [45] were performed as described previously. For all stages, gonad-only samples (mesonephroi removed) were used, which were snap-frozen in liquid nitrogen immediately after dissection. For RT-qPCR, 200 ng of input RNA, pooled from like samples, was subjected to cDNA synthesis with SuperScript III First-Strand Synthesis System for RT-PCR (Invitrogen) as per manufacturer’s instructions. 1μl of the resultant cDNA reaction was used in a 20μl qPCR mastermix containing 1 SYBR Green PCR Master Mix (Applied Biosystems) and 175nM each of the forward and reverse primers. 5μl triplicate reactions were run in 384-well plates on a Viia7 Real Time PCR System (Applied Biosystems) as technical replicates. The PCR products were analyzed by gel electrophoresis, cloned and sequenced to verify specificity of amplified sequence, and primer efficiency was determined. Gene expression was normalized to *Sdha* [43]. For ddPCR, cDNA samples were diluted with RNAse-free water 1:10 to 1:1000 for expression analysis. ddPCR was performed using a BioRad QX100 system. A two-step thermocycling protocol [95°C, 10 min; 40× (94°C, 30 s, 60°C, 60 s); 98°C, 10 min; ramp rate set at 2.5°C/s] was carried out in a BioRad C1000 Touch thermal cycler. Analysis of the ddPCR data was performed with QuantaSoft analysis software (BioRad). ddPCR data were normalized to *Tbp* [43].

RT-PCR analyses were performed on at least three independent biological samples. For each gene, data sets were analyzed for statistically significant differences between XX and XY expression levels using a two-tailed, unpaired t-test with confidence intervals set at 95%. Primers used are described in **S1 Table**.

### Patient case reports

Patient 1 from Poland was diagnosed at the age of 16 years with 46,XY DSD with gonadal dysgenesis. The patient displayed primary amenorrhea, female genitalia, undeveloped secondary sexual characteristics, small hypoplastic uterus, gonadal dysgenesis, streak gonads, the height of 175cm, and a 46,XY karyotype. Prolactin was at normal level, FSH et 80 IU/L, LH at 50 IU/L and low estradiol levels at 5pg/mL. Genomic DNA from the proband was isolated from blood samples using the Qiagen DNA mini kit (Qiagen, Valencia, California).

Patient 2 from Indonesia was identified as 46,XY with suspected gonadal dysgenesis and severe hypospadias. The patient was Quigley stage 2 with a phallus of 2cm and the urethral meatus located in scrotal area. Chordee was also noted. The right testicle (2ml in volume) was present in the right scrotal region, while the left testis was absent and could not be detected by ultrasound.

### Massively parallel sequencing

Patient recruitment, consent and DNA extraction was carried out described previously [7]. Total genomic DNA was sequenced on a targeted panel (HaloPlex, Agilent) that includes 64 diagnostic DSD genes (described in [7]).

### Exome sequencing, comparative genomic hybridization (CGH) and qPCR

An array-CGH 244K (Agilent) was performed using the manufacturer's recommended protocols without modifications. Exome capture was performed using the SureSelect Human All Exon v3 kit (Agilent Inc). Sequencing was carried out on an Illumina HiSeq 2000 instrument. Fastq files were obtained using the Illumina CASAVA v1.8.1 software and processed using our “in house” bioinformatic pipeline running on the Vital-IT Center for high-performance computing of the Swiss Institute of Bioinformatics (SIB; http://www.vital-it.ch) as described in [46]. Results did not reveal obvious pathogenic variants. The detected deletion was confirmed by quantitative PCR on genomic DNA using the primers listed in **S1 Table**.

### Generation of wildtype and mutant *HMGCS2* expression constructs

cDNA encoding *HMGCS2* protein without the signal peptide was amplified from liver by PCR and cloned into the expression plasmid pMAL-c2x as described in [26]. Variant c.1502G>C (p.Arg501Pro) was introduced on pMAL-*HMGCS2* using the QuickChange^™^ Site-Directed Mutagenesis Kit (Agilent) according to the manufacturer’s instructions. DNA sequencing of the new construct was performed to confirm target mutation.

### Expression and purification of wildtype and mutant HMGCS2 proteins

*E*. *coli* strain BL21 expressing MBP-HMGCS2 wild-type or mutant protein were grown in LB medium (10 g Peptone 140, 5 g Yeast Extract, 5 g sodium chloride per liter) at 37°C to an A_600_ of 0.8–1.0. Optimal protein expression was induced with 0.3mM IPTG at 20°C for 18h. Cells were recovered, lysed and disrupted by thermal shock at 37ºC (15min), 80ºC (45min) and 37ºC (3min). The soluble fraction containing the MBP-HMGCS2 fusion proteins were loaded into an amylase affinity column, washed, and finally eluted from the affinity resin using a buffer containing the protease factor Xa [26].

### Western blot analysis

Purified proteins were quantified by Bradford’s method. 5μg of each protein sample were subjected to 15% SDS-PAGE electrophoresis and transferred to a 0.45μm PVDF membrane. Membranes were probed with a 1:500 dilution of a monoclonal antibody (M06) against amino acids 424–508 of human HMGCS2 (Abnova, Taipei City, Taiwan) and a 1:1000 dilution of a secondary anti-mouse antibody. The blots were developed with the Immobilon Western Chemiluminescent HRP Substrate (Millipore) kit. The images obtained were processed using Adobe Photoshop 5.0. The relative amounts of mutated compared with wildtype (assigned 100%) HMGCS2 protein were determined using the software “Image Studio Lite Analysis Software for Western Blots”.

### Enzymatic activity

Mitochondrial HMG-CoA synthase activity was determined measuring the amount of acetoacetyl-CoA by spectrophotometry at 304 nm, as previously described in the literature. Each experiment was performed in triplicate [26].

### *In silico* analyses

For evolutionary sequence comparisons the following NCBI (https://www.ncbi.nlm.nih.gov) reference proteins were used: *Homo sapiens* HMGCS2 (NP_005509.1), *Mus musculus* HMGCS2 (NP_032282.2), *Bos taurus* HMGCS2 (NP_001039348.1), *Homo sapiens* HMGCS1 (NP_001091742.1), *Mus musculus* HMGCS1 (NP_666054.2), *Bos taurus* HMGCS1 (NP_001193507.1), *Danio rerio* HMGCS (NP_957379.2), *Drosophila melanogaster* HMGCS (NP_524711.1), and *Arabidopsis thaliana* HMGCS (NP_192919.1). The reference proteins were aligned with the online program Clustal Omega (https://www.ebi.ac.uk/Tools/msa/clustalo/). To predict the effect of the substitution p.R501P on HMGCS2 protein function the following *in silico* algorithms were used: PolyPhen-2 (http://genetics.bwh.harvard.edu/pph2/) [47], SIFT (http://sift.jcvi.org/) [48], and MutationTaster (http://www.mutationtaster.org/) [49]. The PyMOL molecular visualization system (Schrödinger) was used for computational modeling of the p.Arg501Pro substitution based on the human HMGCS2 protein (PDB 2WYA [50]).

## Results

### *Hmgcs2* switches from testis to ovary-enriched expressed during fetal gonad development

**Fig 1:**
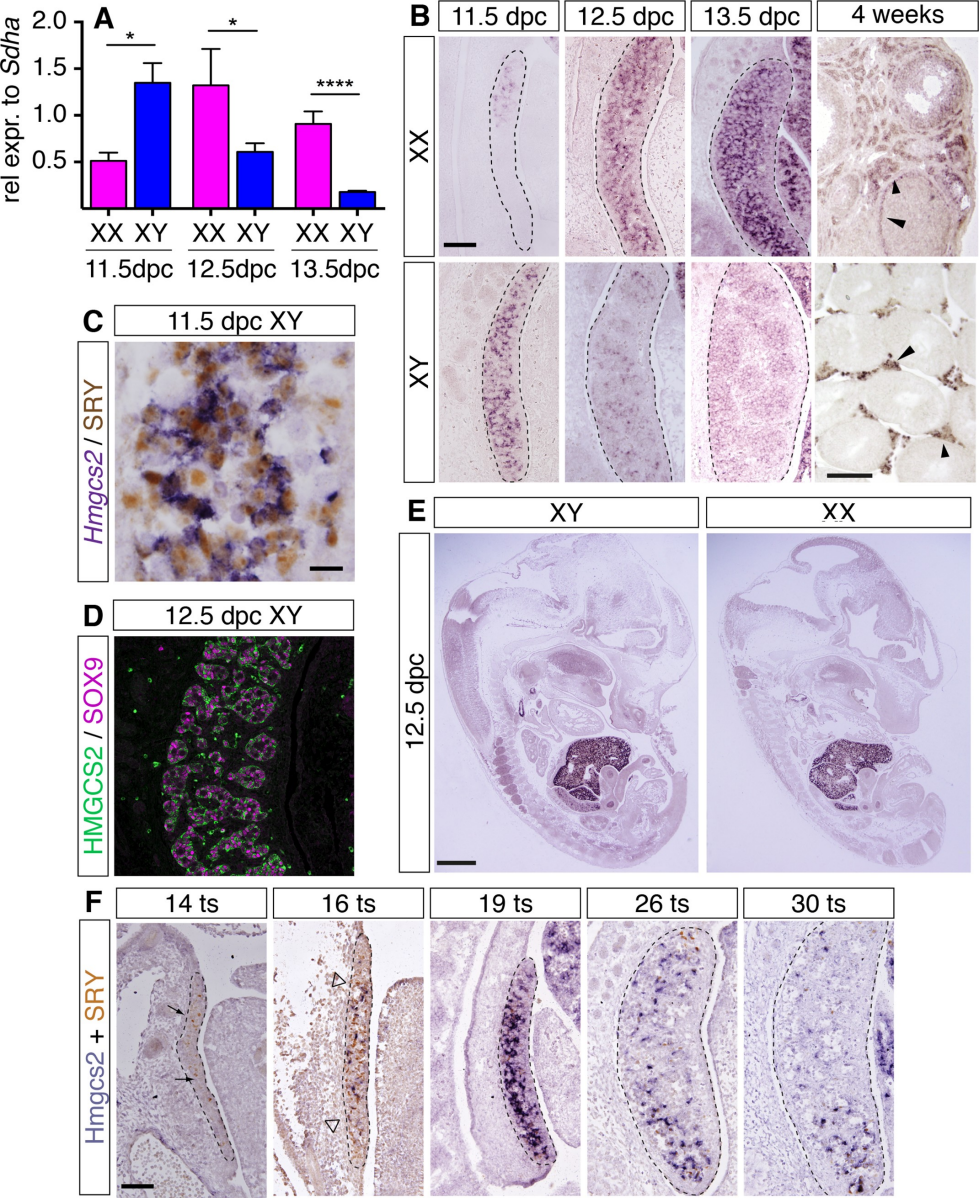
*Hmgcs2* expression switches from testis-to-ovary enriched. (**A**) qRT-PCR analyses for *Hmgcs2* in XY (blue bars) and XX (pink bars) gonads between 11.5 and 13.5 dpc. Mean ± SEM, n = 3. *P < 0.05, ****P < 0.0001. (**B**) Section ISH for the *Hmgcs2* transcript on XY and XX gonad sections between 11.5 and 13.5 dpc (scale bar, 100 μm), and at 4 weeks (scale bar, 30 μm). (**C**) *In situ* hybridization for the *Hmgcs2* transcript (purple), followed by immunohistochemistry for SRY (brown) on XY gonad sections between 14 and 30 tail somites. Scale bar, 15 μm. (**D**) Double immunofluorescence for HMGCS2 (green) and the Sertoli cell marker SOX9 (purple) on testis sections at 12.5 dpc. Scale bar, 50 μm. (**E**) Section ISH for *Hmgcs2* on whole XY and XX embryos at 12.5dpc. Scale bar, 1 mm. (**F**) Section ISH for the *Hmgcs2* transcript (purple), followed by immunohistochemistry for SRY (brown) on XY gonad sections between 14 and 30 tail somites. Scale bar, 100 μm. All images of fetal gonad sections are oriented so that the anterior pole is at the top and the mesonephros is on the left of the gonad.

In a recent study to identify novel genes that are expressed at higher levels in the ovary compared to testis during early mouse gonad development [38], we discovered that the gene encoding the metabolic enzyme HMGCS2 is ovary-enriched expressed at 13.5 dpc. To analyze in more detail *Hmgcs2* expression during gonad development we used quantitative real-time RT-PCR (RT-qPCR) and section *in situ* hybridization (ISH) of mouse gonads at different stages during fetal development and postnatally (**Fig 1**). RT-qPCR analyses showed that at 11.5dpc *Hmgcs2* was expressed almost 3-fold higher in XY than in XX gonads (**Fig 1A)**. However, at 12.5dpc *Hmgcs2* expression had decreased in XY gonads, while at the same time expression had increased in XX gonads, resulting in approximately 2-fold higher *Hmgcs2* expression levels in XX compared to XY gonads (**Fig 1A)**. At 13.5dpc, *Hmgcs2* expression in XY gonads continued to be down-regulated and was approximately 5-fold lower than in XX gonads (**Fig 1A)**. We confirmed these findings by section ISH analysis. At 11.5dpc, *Hmgcs2* was strongly expressed throughout the XY gonad, but was down-regulated at 12.5dpc and was barely detectable by 13.5dpc (**Fig 1B**). In contrast in XX gonads, *Hmgcs2* was only weakly expressed at 11.5dpc, but was up-regulated at 12.5dpc and continued to be strongly expressed at 13.5dpc (**Fig 1B**).

The pattern of expression as seen in the section ISH suggested that *Hmgcs2* was expressed in somatic cells but not in germ cells. To confirm the expression in somatic cells, we performed section ISH for the *Hmgcs2* transcript (purple staining) followed by immunohistochemistry (IHC, brown staining) for the SRY protein, which is known to be expressed in pre-Sertoli cells at 11.5dpc [51]. This analysis demonstrated that the *Hmgcs2* gene was expressed in SRY-positive pre-Sertoli cells, although not all SRY-positive cells were also *Hmgcs2*-positive (**Fig 1C**). One day later, at 12.5dpc, double immunofluorescence (IF) analysis for HMGCS2 and the Sertoli cell marker SRY-box 9 (SOX9) confirmed that HMGCS2 was expressed in Sertoli cells of the developing testis cords (**Fig 1D**). In contrast, at 4 weeks of age, *Hmgcs2* expression was detected in steroidogenic cells, theca and Leydig cells in ovaries and testes respectively (**Fig 1B**, right panel), as has been described before [31]. Furthermore, ISH of sections of whole embryos at 12.5dpc showed that the main sites of *Hmgcs2* expression were the developing liver and gonads (**Fig 1E).** Taken together, these data confirmed that *Hmgcs2* is enriched in XY gonads at the time of testis determination and that its expression switches from testis to ovary-enriched between 11.5dpc and 12.5dpc.

To explore in more detail the spatio-temporal relationship between *Hmgcs2* and SRY expression in the developing testes, we performed expression analysis between 11.0dpc and 12.5dpc by counting tail somites (ts), a more accurate staging system [33]. As previously shown [39], SRY was only detected in a few scattered cells in the central region of the genital ridge at 14ts (**Fig 1F**, first panel, brown staining), but was robustly expressed throughout the XY genital ridges by 16ts (**Fig 1F**, second panel). In contrast, *Hmgcs2* expression was not yet detectable at 14ts (**Fig 1**, first panel) and was only observed in a few cells at 16ts (**Fig 1F,** second panel, purple staining). The *Hmgcs2*-positive cells were, comparable to SRY-positive cells at 14ts, predominantly located in the central region of the developing testis (**Fig 1F**, second panel). By 19ts, *Hmgcs2* was strongly expressed throughout the XY genital ridges (**Fig 1F**, third panel). After 19ts, both the number of SRY- and *Hmgcs2*-expressing cells declined, and they were mainly restricted to the gonadal poles by 26ts and 30ts (**Fig 1F**, fourth and fifth panel). These data showed that expression of *Hmgcs2* closely follows that of SRY with a delay of approximately 2 tail somites, equaling around 4h [52], during the critical phase of gonadal sex determination.

### *Hmgcs2*-null mice develop normally

**Fig 2:**
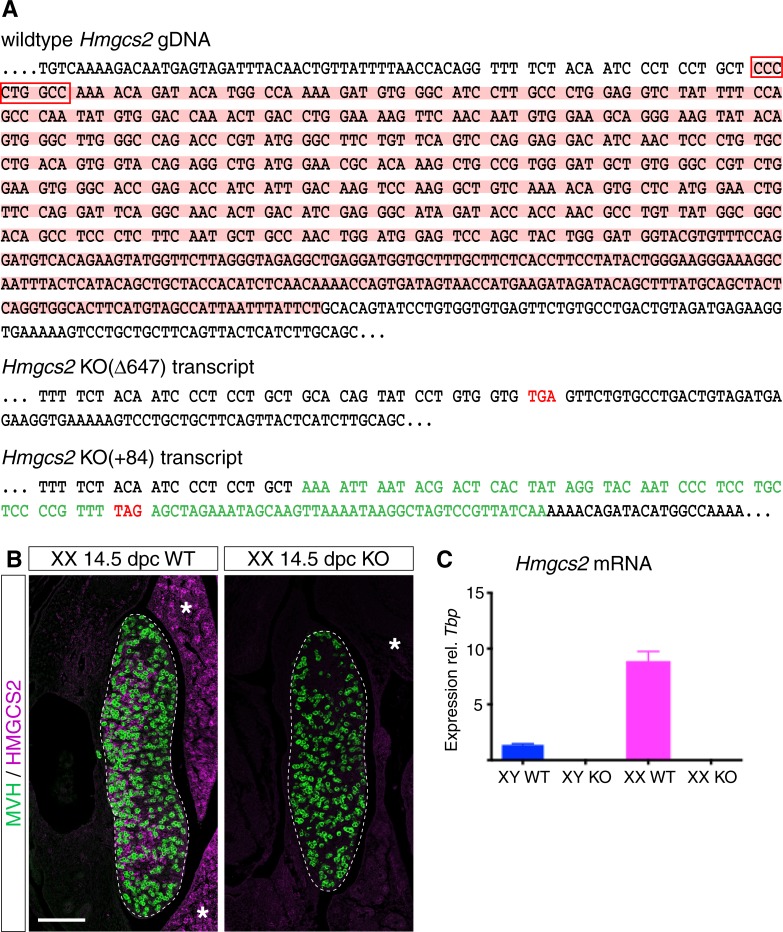
Generation of *Hmgcs2*-null mice. (**A**) Part of the genomic DNA sequence of the mouse *Hmgcs2* gene. Exon 2, which was targeted with CRISPR/Cas9 genome editing, is marked in bold with codons separated by blanks. Two stable mouse lines were generated with founder 1 (*Hmgcs2* KO(Δ647) transcript) having a deletion of 647 nucleotides (marked with pink underlay in upper panel) encompassing most of exon 2 and part of intron 2, resulting in a premature stop codon (marked in red in middle panel). Founder 2 (*Hmgcs2* KO(+84) transcript) had a deletion of 9 nucleotides (red box in upper panel) in addition to an insertion of 93 nucleotides (marked in green in bottom panel), also resulting in a premature stop codon (marked in red in bottom panel). (**B**) Double immunofluorescence analysis for HMGCS2 (purple) and MVH (green) on sagittal sections of wildtype (WT) and *Hmgcs2*-null (Δ647 KO) mouse fetuses at 14.5 dpc shows loss of HMGCS2 protein in the knockout. Scale bar, 100 μm. All images of fetal gonad sections are oriented so that the anterior pole is at the top and the mesonephros is on the left of the gonad. Asterisks mark liver. (**C**) Quantitative ddRT-PCR for *Hmgcs2* mRNA in 14.5 dpc testes (blue bars) and ovaries (pink bars) from wildtype (WT) and *Hmgcs2*-null (Δ647 KO) mice demonstrated absence of *Hmgcs2* exon 2 in the knockout. ddRT-PCR data were obtained from at least three independent samples. Error bars represent SEM.

To functionally test the role of *Hmgcs2* in gonad development *in vivo* we used CRISPR/Cas9 genome editing to generated mice lacking a functional HMGCS2 protein. A CRISPR guide RNA (**S1 Table**) was designed to target exon 2 of the *Hmgcs2* gene. Sequence analysis identified sixteen indels ranging from -647 to +84 nucleotides in 19 out of 25 offspring. Among the 19 mutated animals, 8 were compound heterozygotes, 3 were heterozygous and 8 were mosaic. We generated lines for two of the founders with the first one having a deletion of 647 nucleotides (named Δ647) spanning from the start of exon 2, which encodes two of three amino acids (Glu132 and Cys166) of the catalytic triad [50], into intron 2 (**Fig 2A**, upper panel, pink underlay), resulting in a stop codon seven codons after the break point (**Fig 2A**, second panel). The second founder (named +84) had a deletion of 9 nucleotides within exon 2 of the *Hmgcs2* gene (**Fig 2A**, red box), in addition to an insertion of 93 nucleotides of largely unrelated sequences (**Fig 2A**, sequence in green, bottom panel), which also resulted in a premature stop codon (**Fig 2A**, bottom panel). For both lines, mice heterozygous for the mutation were viable and fertile, and were subsequently used to generate homozygous *Hmgcs2*^-/-^ embryos. Both lines had the same phenotype, hence only data for the (Δ647) is shown.

First, we tested if HMGCS2 was successfully deleted in these mice. IF analysis using an HMGCS2-specific antibody (**Fig 2B**, purple) together with a marker for germ cells, MVH (**Fig 2B**, green) on paraffin sections of wildtype and *Hmgcs2*-null ovaries at 14.5 dpc demonstrated that in contrast to wildtypes, HMGCS2 protein was undetectable in the ovary and liver in the knockout animals (**Fig 2B**). Quantitative ddRT-PCR on RNA isolated from 14.5dpc wildtype and knockout testes and ovaries confirmed that exon 2 is missing in *Hmgcs2*-null mice (**Fig 2C**).

**Fig 3:**
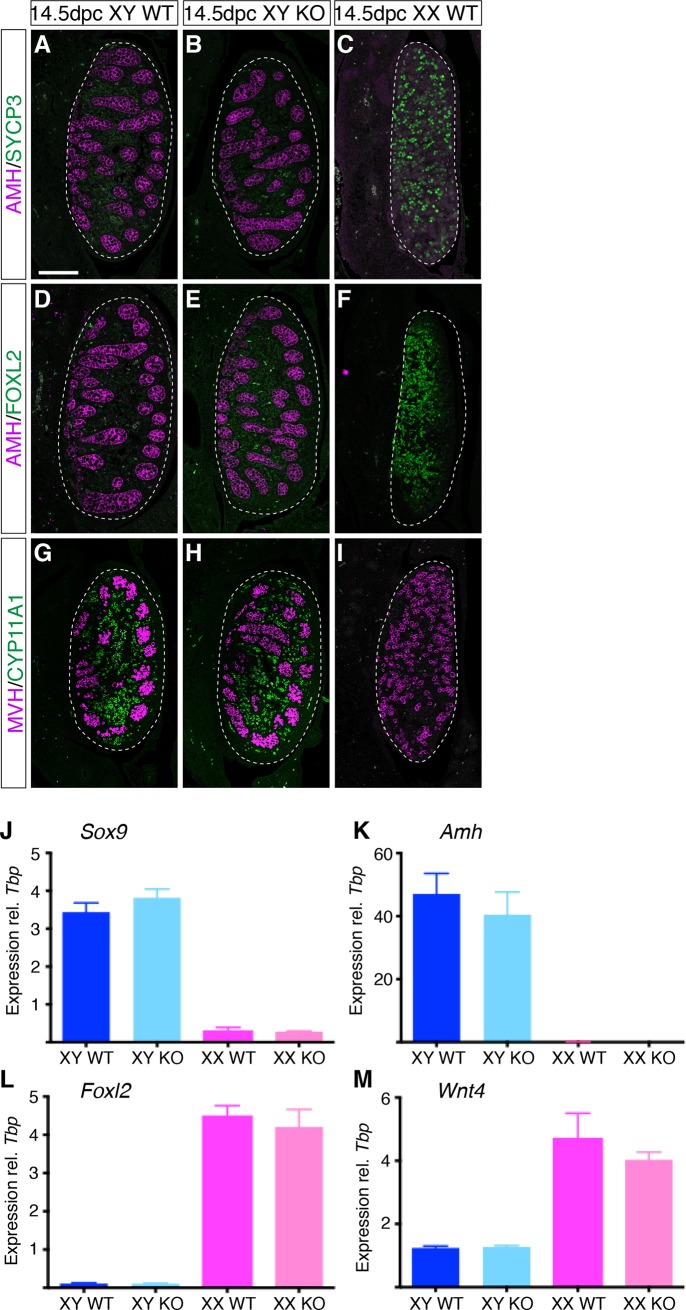
Markers of gonadal differentiation in wildtype and *Hmgcs2*-null mice. (**A-I**) Double immunofluorescence on sagittal sections of gonads from wildtype (XY and XX WT) and *Hmgcs2*-null (XY KO (Δ647)) fetuses at 14.5 dpc for (**A-C**) AMH (purple, Sertoli cells) and SYCP3 (green, germ cell meiosis); (**D-F**) AMH (purple) and FOXL2 (green, pre-granulosa cells); and (**G-I**) MVH (purple, germ cells) and CYP11A1 (green, Leydig cells). Scale bar, 100 μm. All images of fetal gonad sections are oriented so that the anterior pole is at the top and the mesonephros is on the left of the gonad. (**J-M**) Expression of mRNA was measured at 14.5 dpc by quantitative ddRT-PCR and is shown relative to the expression levels of *Tbp*. XX samples are shown in pink, XY in blue. (**J**) *Sox9* expression; (**K**) *Amh*; (**L**) *Foxl2*; (**M**) *Wnt4*. ddRT-PCR data were obtained from at least three independent samples. Error bars represent SEM.

In order to determine if loss of HMGCS2 affects testis determination and development, we performed double IF on paraffin sections of wildtype and *Hmgcs2*-null embryos at 14.5 dpc. All XY *Hmgcs2*-null embryos developed testes that were indistinguishable from wildtype testes as shown by the formation of testes cords (compare **Fig 3B, 3E and 3H** with **Fig 3A, 3D and 3G**), the expression of the Sertoli cell marker AMH (**Fig 3A** and **3B**) and Leydig cell marker CYP11A1 (**Fig 3G and 3H**), as well as the absence of the meiosis marker SYCP3 (**Fig 3B and 3C**) and the granulosa cell marker FOXL2 (**Fig 3E and 3F**), which are ovary-specific at this stage. Furthermore, quantification of testicular and ovarian markers by quantitative ddRT-PCR confirmed that XY *Hmgcs2*-null testes expressed *Sox9* (**Fig 3J**, blue bars) and *Amh* (**Fig 3K**, blue bars) at similar levels to those seen in wildtype XY testes, and at much higher levels than in XX wildtype and XX *Hmgcs2*-null gonads (**Fig 3J and 3K**, pink bars). Also in agreement with the IF analysis, *Foxl2* expression levels in XY wildtype and *Hmgcs2*-null gonads were negligible (**Fig 3L**, blue bars), in contrast to wildtype and *Hmgcs2*-null XX gonads (**Fig 3L**, pink bars). In addition, the mRNA levels of another ovarian marker, *Wnt4*, in XY *Hmgcs2*-null gonads were comparable to those of XY wildtype and markedly lower to the expression levels in XX gonads of either genotype (**Fig 3M**).

Similar to XY animals, XX *Hmgcs2*-null mice developed ovaries indistinguishable from XX wildtype (**S1 Fig**), including normal differentiation of somatic supporting cells, shown by the expression of FOXL2 (**S1A–S1C Fig**, green fluorescence), and germ cells, shown by the meiosis marker SYCP3 (**S1D–S1F Fig**, green fluorescence) at 14.5 dpc. Furthermore, testicular markers such as SOX9 were not expressed in 14.5 dpc XX *Hmgcs2*-null gonads (**S1G–S1I Fig**, green fluorescence). Quantification of gonadal markers by quantitative ddRT-PCR demonstrated that XX *Hmgcs2*-null ovaries expressed the ovarian markers *Foxl2* (**Fig 3L**, pink bars) and *Wnt4* (**Fig 3M**, pink bars) at similar levels to those seen in XX wildtype ovaries. In addition, the mRNA levels of the testicular markers *Sox9* (**Fig 3J**, blue bars) and *Amh* (**Fig 3K**, blue bars) in XX *Hmgcs2*-null ovaries were very low compared to XY wildtype and *Hmgcs2*-null gonads and similar to wildtype XX ovaries.

These data show that in mouse, gonad development and differentiation are not affected by the loss-of-function of HMGCS2.

### Gonads develop normally in *Hmgcs2*^-/-^:*Fgr2c*^-/+^ mice

**Fig 4:**
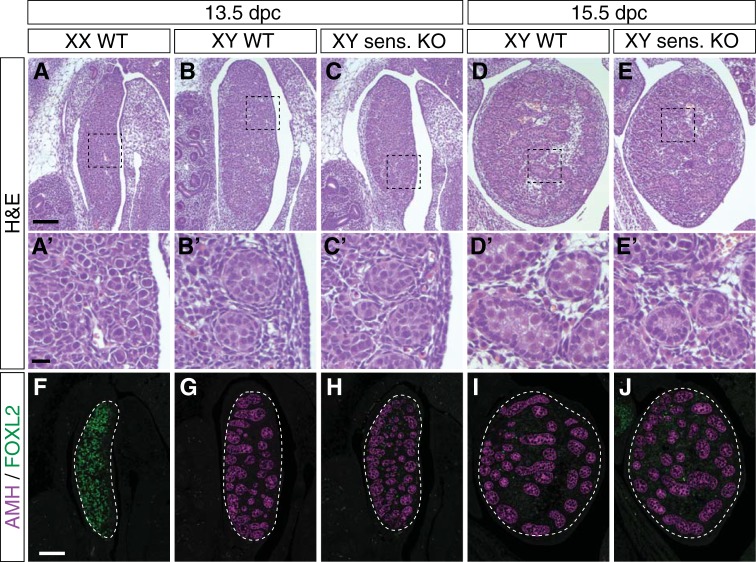
Histology and immunofluorescence analysis of *Hmgcs2*^-/-^:*Fgfr2c*^-/+^ testes. H&E staining (**A-E**) and double IF analysis (F-J) on sagittal sections of paraffin-embedded 13.5 dpc XX (**A, A”, F**) and XY (**B, B’, G**) wildtype, 13.5 dpc XY *Hmgcs2*^-/-^:*Fgfr2c*^-/+^ (sens. KO, **C, C’, H**), as well as 15.5 dpc XY wildtype (**D, D’, I**) and *Hmgcs2*^-/-^:*Fgfr2c*^-/+^ (sens. KO, **E, E’, J**) gonads showed normal testicular morphology in *Hmgcs2*-null on the sensitized background. (**A’-E’**) Enlargement of the regions marked with a rectangle in the upper panel (**A-E**). Scale bars, 100 μm (**A-E**); 30 μm (**A’-E’**), 100 μm (**F-J**). All images of whole fetal gonad sections are oriented so that the anterior pole is at the top and the mesonephros is on the left of the gonad.

A gonadal phenotype in mice might only be evident on a sensitized background; hence we crossed the *Hmgcs2*-null (+84) onto a *Fgfr2c* heterozygous background. FGFR2c has been shown to be important for testis development; likely through the repression of the WNT4- and FOXL2-driven ovarian determining pathways [53]. While XY *Fgfr2c* heterozygous mice develop normal testes, XY *Fgfr2c*-null mice display complete testis-to-ovary sex reversal by 15.5 dpc [53]. Histological analysis using hematoxylin and eosin staining (H&E) on paraffin sections of XX and XY wildtype controls (**Fig 4A, 4B and 4D**; WT) as well as XY compound mutant *Fgfr2c*^-/+^:*Hmgcs2*^-/-^ (**Fig 4C and 4E**; sensitized KO) at 13.5 and 15.5 dpc demonstrated that mice heterozygous for *Fgfr2c* and homozygous null for *Hmgcs2* developed testes that appeared normal compared to the wildtype control, with testis cords throughout at 13.5 dpc and 15.5 dpc (**Fig 4A–4E**). In addition, double IF analysis for the Sertoli cell marker AMH and the granulosa cell marker FOXL2 on sections of 13.5 dpc XX and XY wildtype and XY *Hmgcs2*-null (**Fig 4F–4H**), as well as 15.5 dpc XY wildtype and *Hmgcs2*-null fetuses (**Fig 4I and 4J**) demonstrated that also at the molecular level XY *Hmgcs2*-null mice developed normal testes.

### Identification of a 20 kb deletion within the *HMGCS2* gene in a 46,XY DSD patient with complete gonadal dysgenesis

**Fig 5:**
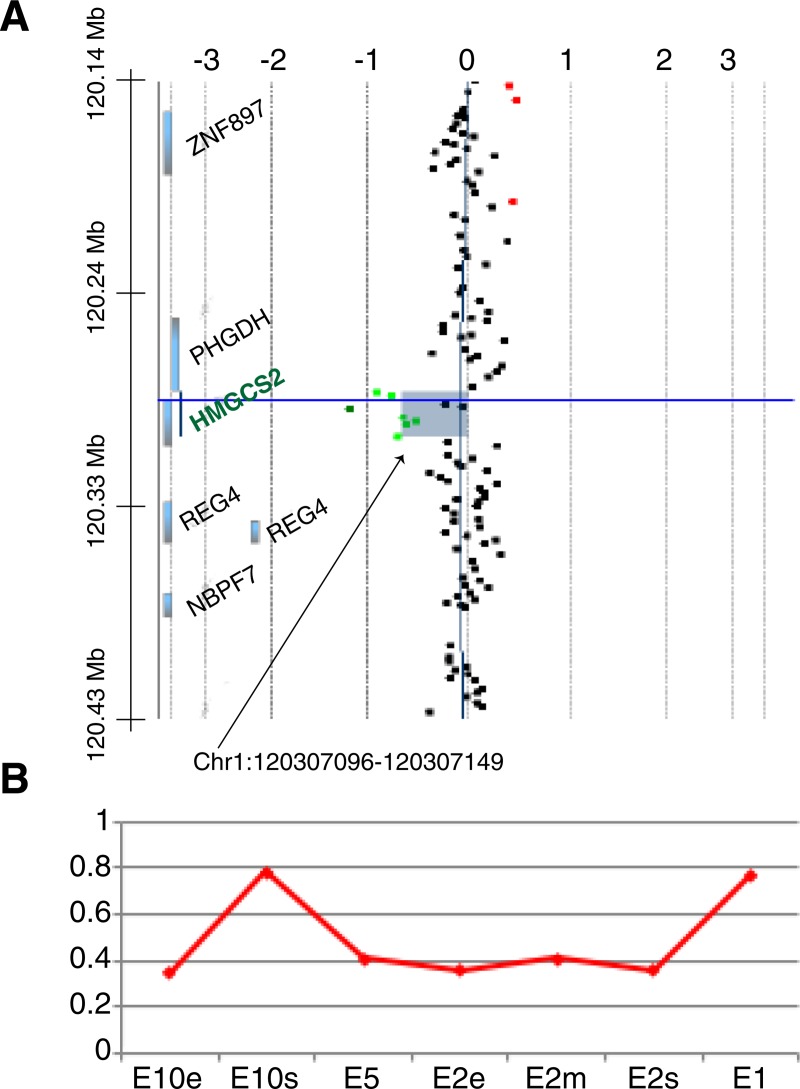
Identification of a heterozygous deletion in the *HMGCS2* gene in a 46,XY GD patient. (**A**) Array comparative genomic hybridization (CGH) identifies a heterozygous deletion of approximately 20 kb (in green) within the *HMGCS2* gene from exon 2 to 10 in patient 1. Chromosomal arrangement on the left. x axis, gene copy number with 0 = 2 copies. (**B**) qPCR analyses validated the deletion up to exon 5. x-axis: primer pairs; E, exon; s, at start of exon; m, in the middle of exon; e, at the end of exon.

To test if HMGCS2 plays an important role in gonad development in humans we first used 1 million probe comparative genomic hybridization arrays (aCGH) on genomic DNA from twenty-three unexplained cases of 46,XY GD European descent, for which variants in *SRY* and in other genes known to cause 46,XY DSD, such as nuclear receptor subfamily 5, group A, member 1 (*NR5A1*) and mitogen-activated protein kinase kinase kinase 1 (*MAP3K1*) [6, 54, 55], were excluded. Among them, we identified a heterozygous deletion within the *HMGCS2* gene in a 46,XY DSD patient with complete gonadal dysgenesis and male-to-female sex reversal (**Fig 5A**). The deletion had a size of approximately 20 kb and removed almost the entire *HMGCS2* gene, from exon 2 until the last non-coding exon 10 (**Fig 5A**). Quantitative PCR analyses on the patient’s genomic DNA validated the deletion from exon 2 to at least exon 5 (amino acids 35 to 339) of the *HMGCS2* gene (**Fig 5B**). Any protein generated from the shortened *HMGCS2* transcript would lack all three amino acids (Glu132, Cys166, and His301; [50] of the catalytic triad, rendering the truncated protein non-functional. As DNA samples from family members were unavailable, it is unknown if the deletion is *de novo* or if any family member displayed any clinical signs or symptoms.

### Identification of a heterozygous *HMGCS2* missense variant in a 46,XY DSD gonadal dysgenesis patient

**Fig 6:**
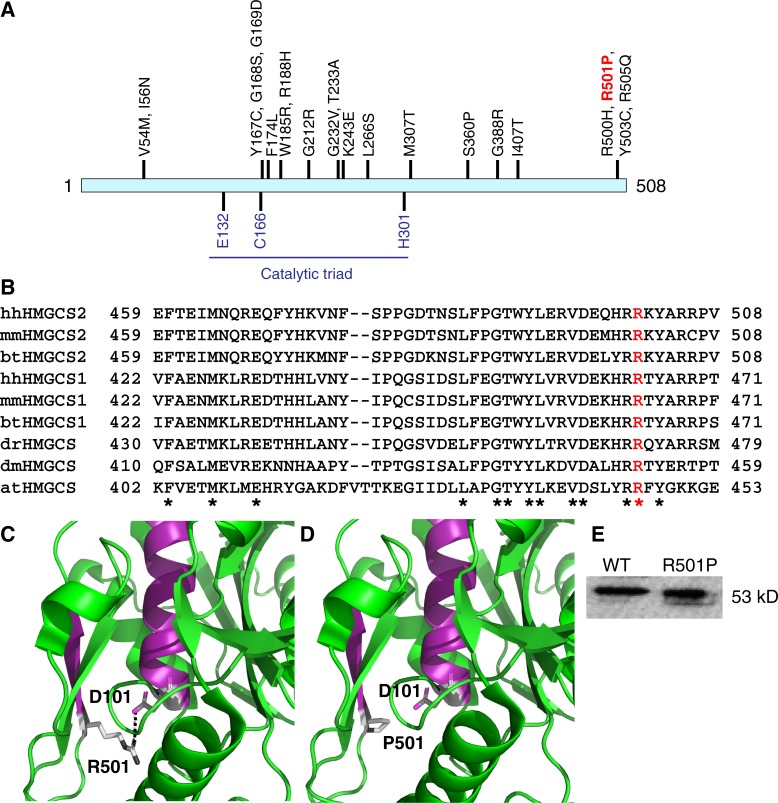
Identification of a heterozygous *HMGCS2* missense variant c.1502G>C (p.Arg501Pro) in a 46,XY GD patient, predicted to disturb protein structure. (**A**) The substitution p.Arg501Pro (red) is located close to the C-terminus of the 508 amino acid HMGCS2 protein. Previously reported missense variants identified in HMGCS2 deficiency patients [25] are indicated above the HMGCS2 protein. The catalytic triad, which consists of the three amino acids glutamic acid 132 (Glu132), cysteine 166 (Cys166) and histidine 301 (His301) [50], is shown below the HMGCS2 protein. (**B**) Evolutionary sequence comparison using Clustal Omega shows that arginine 501 (R, in red) is highly conserved in the mammalian HMGCS2 proteins and in HMGCS1 homologs. The accession numbers for the reference proteins used can be found in the Methods section. hh, *Homo sapiens*; mm, *Mus musculus*; bt, *Bos taurus*; dr, *Danio rerio*; dm, *Drosophila melanogaster*; at, *Arabidopsis thaliana*. ‘*’, identical amino acids. (**C, D**) Computational modeling of the p.Arg501Pro substitution based on the human HMGCS2 protein structure (PDB 2WYA [50]) using the PyMOL molecular visualization system predicts loss of a salt bridge, which is normally formed (dotted line) between arginine 501 (R501) of beta-sheet E17 (in purple) and aspartic acid 101 (D101) of helix H3 (also in purple). (**E**) Western blot of purified wildtype (WT) and variant (Arg501Pro) proteins.

To search for additional 46,XY GD cases with variants in the *HMGCS2* locus, we incorporated the *HMGCS2* gene into our targeted massively parallel sequencing (MPS) screen, which offers a tool for diagnosis and for identifying novel DSD genes [7]. The HaloPlex screen (Agilent) currently has a targeted region size of 2.5 Mb covering 1031 genes, including all known DSD genes as well as candidate DSD genes. Among 52 patients with 46,XY GD that were sequenced by MPS, we identified a previously unreported, heterozygous *HMGCS2* missense variant in exon 9, c.1502G>C (p.Arg501Pro) in a 46,XY GD patient (**Fig 6A**). Family members of this patient were not available for sequencing.

The change affects two transcripts, both of which are expressed in the testis: NM_005518: c.1502G>C; p.(Arg501Pro) and NM_001166107: c.1437G>C; p.(Arg459Pro). The variant is predicted to result in a major amino acid change from arginine to proline (Grantham score: 103). All of the *in silico* protein predictions deemed the change to be pathogenic (PolyPhen, Mutation taster and SIFT). Although the variant is present in gnomAD; it is extremely rare (reported in 0.0012%, 0 homozygotes), however its slightly more prevalent in the “Other East Asian” population (frequency: 0.011%, 0 homozygotes). It should be noted that our patient is from a South Asian origin. We could not establish whether the variant was *de novo* as the parents were not available for analysis. There is also a ClinVar entry which describes the variant seen in our patient and concludes that it is a variant of uncertain significance. In addition to the variant identified in our patient, there are a further two other variants that have been reported in gnomAD. A rare missense change c.1502C>T;p.Arg501Lys (frequency 0.00039%, 0 homozygotes) as well as a premature stop codon c.1501G>A; p.Arg501Ter (frequency: 0.0012%, 0 homozygotes).

To test if p.Arg501Pro could affect HMGCS2 protein structure, we first performed *in silico* analyses. While the mitochondrial HMGCS2 enzyme is restricted to mammals, its homolog, the cytosolic HMGCS1 enzyme, is highly conserved in eukaryotes including frog, zebrafish, fruit fly, and plants. Evolutionary sequence comparison revealed that arginine 501 (Arg501) is highly conserved in mammalian HMGCS2 and HMGCS1 proteins as well as in HMGCS1 homologs (**Fig 6B**), suggesting that this amino acid is critical for HMGCS protein function. Consistent with this, human p.Arg501Pro was predicted to be damaging by the three *in silico* algorithms SIFT (score 0) [48], MutationTaster (score 0.99) [49], and PolyPhen-2 (score 1) [47] with near-maximal to maximal scores. Based on the human HMGCS2 protein structure (PDB 2WYA [50]), it was predicted that the positively charged Arg501 of beta sheet E17 forms a salt bridge with the negatively charged aspartic acid 101 (Asp101) of helix H3 near the dimerization surface (**Fig 6C**). If arginine is replaced by the non-polar proline at position 501 this salt bridge can no longer form and therefore is likely to disturb protein structure (**Fig 6D**). In addition, Arg501 is located within beta sheet E17 (**Fig 6C**) and proline is a known beta sheet breaker [56], suggesting that p.Arg501Pro will disrupt the beta sheet and therefore the protein structure.

To confirm the *in silico* results of the variant HMGCS2 protein in a functional assay, we expressed wildtype and variant HMGCS2 proteins tagged with MBP in *E*. *coli* and purified them using amylose affinity columns [26]. Western blot analysis showed that the mutant protein was obtained in a soluble form and at a similar level (89.3% of that of wild-type) (**Fig 6E**). Next, we measured HMGCS2 enzymatic activity using a spectrophotometric method that determines the amount of acetoacetyl-CoA consumed [26]. In contrast to the wildtype HMGCS2 protein, the variant HMGCS2 protein showed no detectable specific activity (**Table 1**; n = 3). This demonstrates that the missense variant c.1502G>C (p.Arg501Pro) completely abolished HMGCS2 enzymatic activity. In summary, although the functional data presented here supports the importance of this residue, following ACMG guidelines the current evidence is insufficient to conclude that this variant is having a clear role in the patient’s phenotype and therefore we have classified it as a variant of uncertain significance.

**Table 1:** Enzymatic activity of purified wildtype and variant HMGCS2. Specific HMGCS2 enzymatic activity of wildtype and variant (Arg501Pro) was measured using a spectrophotometric method that determines the amount of acetoacetyl-CoA consumed.

Variation	Exon	Protein effect	Specific activity (μmol/min.mg-enz)	% Activity
WT	-	-	0.910±0.01	100%
c.1502G>C	E9	p.R501P	nd	0%

nd—not detectable

In summary, while *Hmgcs2* shows a unique expression pattern in mouse fetal gonads, which implies it might be necessary for sex differentiation, based on our functional data in mouse and the fact that the two cases of 46,XY DSD with variants in HMGCS2 identified here were heterozygous variants, we believe that it is unlikely that HMGCS2 plays an important role in gonad development and therefore is an unlikely cause of DSDs in human.

## Discussion

Here, we report the unusual expression pattern of the metabolic enzyme HMGCS2 during mouse fetal gonad development. Our analysis showed that in fetal testes *Hmgcs2* is expressed in the same transient spatio-temporal pattern as *Sry* in supporting Sertoli cells with a delay of about 4h. Interestingly, while *Hmgcs2* expression is down-regulated in the developing testis from approximately 11.5dpc onwards, it is up-regulated in the developing ovary, becoming ovary-enriched from 12.5dpc. To our knowledge this is the only gene that shows a switch from testis- to ovary-enriched expression.

To test a possible role for HMGCS2 in gonad development, we followed two lines of investigations, the generation of mice lacking functional HMGCS2 and the screening of DSD patients for variants in *HMGCS2*. Using CRISPR/Cas9 genome editing we successfully established *Hmgcs2*-null mice. However, phenotypic analysis showed that the loss of this enzyme is not sufficient to cause disruption of fetal gonad development. Furthermore, while we identified two unrelated patients with 46,XY gonadal dysgenesis with a deletion and variant in *HMGCS2*, respectively, both variants were heterozygous. Hence, based on the ClinGen tool (https://clinicalgenome.org/), these patients are not expected to present with the disorder HMGCS2 deficiency and it is questionable whether their DSD phenotype was caused by these variants.

Patient 1 carried a heterozygous deletion, which removed almost the entire *HMGCS2* gene, and presented as a phenotypic female with complete gonadal dysgenesis despite having a 46,XY karyotype. Patient 2, displayed severe hypospadias with suspected gonadal dysgenesis, and carried a heterozygous missense variant c.1502G>C (p.Arg501Pro). The missense variant showed complete loss of HMGCS2 enzymatic activity *in vitro*, suggesting that it could play a role in the DSD phenotype. Computational modelling predicts that the p.Arg501Pro substitution disrupts the protein structure close to the dimeric interface due to loss of a salt bridge that is normally formed between Arg501 and Asp101. Alongside the substitutions p.Arg500His, p.Tyr503Cys and Arg505Gln previously identified in HMGCS2-deficiency syndrome [25, 57], p.Arg501Pro is now the fourth substitution found at the C-terminal end of the HMGCS2 protein (**Fig 5A**), highlighting the critical function of this region.

Homozygous variants in human *HMGCS2* are well known to cause the very rare metabolic disorder HMGCS2 deficiency [24] (OMIM: 605911), which is characterized by severe hypoketotic hypoglycemia, encephalopathy, and hepatomegaly that is triggered after extended periods of fasting or after infections [28]. To date, 27 HMGCS2 deficiency patients have been identified including ten males, three females, and 14 patients for which the sex was not reported [24–28, 57–61]. However, gonadal defects have not yet been described in any of these patients who carry homozygous or compound heterozygous *HMGCS2* variants.

Does this mean that HMGCS2 does not play a role in gonad development in humans and that the DSD individuals described here were caused by other genes? Based on our data in mice, this is certainly the most likely interpretation. Further analysis would be necessary to establish the expression of *HMGCS2* in human fetal gonads as well as investigate a larger number of patients to establish an association of *HMGCS2* with gonadal development. However, there are examples of genes, such as the testis-determining gene *Sox9*, for which it has been shown that there is a difference in dosage requirements between human and mouse. In humans, loss of one *SOX9* allele is sufficient to cause XY sex reversal in most cases [8, 9], whereas in mouse both alleles need to be deleted [10, 11, 62]. There is a possibility that the two DSD individuals described here carry modifier genes that exacerbate specifically the testicular phenotype with no influence on the metabolic role of HMGCS2. Indeed, there is recent data suggesting an oligogenic basis for some DSDs [63–65]. It also is possible that the lack of HMGCS2 activity in the developing gonads only affects testis differentiation when combined with low glucose levels at the time of gonadal development (see below), which could be tested by keeping pregnant female mice on a ketogenic diet.

The question remains as to what role HMGCS2 might play during testis determination. HMGCS2 function is best known in the liver where it is important for ßHB production from fatty acids under low glucose conditions, which then can be used as energy source by other organs. In the adult rat testis, HMGCS2 is expressed in steroidogenic Leydig cells, and it was suggested that the expression of HMGCS2 in this cell type is a mechanism for promoting androgen production [31]. In contrast, we show here that in the fetal testis, *Hmgcs2* is not expressed in steroidogenic but in the supporting cell lineage, the Sertoli cells, indicating that HMGCS2 might play a different role during early testis development. We found that *Hmgcs2* is transiently expressed in Sertoli cells at the time of gonadal sex determination, and that its spatiotemporal expression pattern follows closely that of the sex-determining gene *Sry*. The timing of *Hmgcs2* expression correlates with a critical phase during testis determination in which maintenance of SOX9 expression in pre-Sertoli cells and thus Sertoli cell differentiation requires a high-glucose metabolism [14]. It is therefore possible that in humans, if glucose levels are too low, HMGCS2 could provide additional energy from fatty acids required for proper Sertoli cell differentiation.

Recently, it has been demonstrated that apart from providing energy, HMGCS2 also plays an important role in gene regulation [30]. One of the end products of ketogenesis, the ketone body ßHB, is an endogenous and specific inhibitor of class I histone deacetylases (HDACs) [32]. HDACs are a class of enzymes that remove acetyl groups from histones, which is associated with gene silencing. In the intestine, ßHB inhibits HDACs as evidenced by increased acetylation of histone H3 lysine 9 (AcH3K9) to induce the expression of intestinal differentiation markers such as p21 and CDX2 [30]. The production of ßHB by HMGCS2 in the fetal testis could thus be important for the expression of important testicular genes such as *SRY* and *SOX9*, which otherwise would be repressed by HDACs. Indeed, just recently, it has been demonstrated that histone acetylation of the *Sry* promoter is required for *Sry*/SRY expression and thus testis determination [66]. Mouse embryos with reduced histone acetyl-transferase activity showed reduced *Sry* expression and complete XY gonadal sex reversal, most likely due to a reduction in H3K27Ac marks at the *Sry* promoter [66].

In summary, while *Hmgcs2* expression in mouse suggests a role in sex differentiation, current evidence is insufficient to conclude that *HMGCS2* variants could be causative of DSD in humans.

## Supporting information

S1 Fig Markers of gonadal differentiation in XX wildtype and *Hmgcs2*-null mice. Double immunofluorescence on sagittal sections of gonads from XX and XY wildtype (WT) and XX *Hmgcs2*-null (KO (Δ647)) fetuses at 14.5 dpc for (**A-C**) MVH (purple, germ cells) and FOXL2 (green, pre-granulosa cells); (**D-F**) MVH (purple, germ cells) and SOX9 (green, Sertoli cells); and (**G-I**) MVH (purple, germ cells) and SYCP3 (green, meiotic germ cells). Scale bar, 100 μm. All images of fetal gonad sections are oriented so that the anterior pole is at the top and the mesonephros is on the left of the gonad.
(PDF)

S1 Table Primers used in this study. Sequences of primers used in various experiments.
(DOCX)
