# Supplementary figures and images for: The gene encoding the ketogenic enzyme HMGCS2 displays a unique expression during gonad development in mice
Source: PLoS One. 2020 Jan 7;15(1):e0227411. doi: 10.1371/journal.pone.0227411 (PMC6946174; doi:10.1371/journal.pone.0227411)

Figure S1

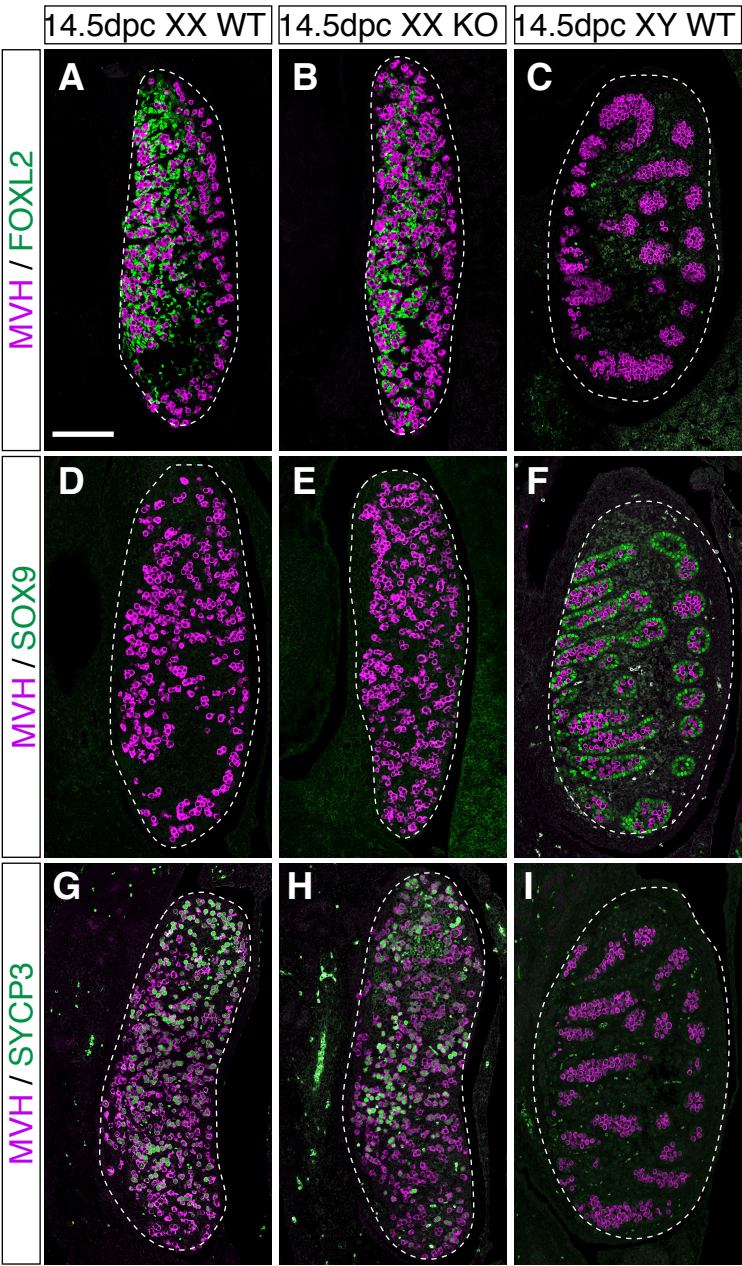

Supplement: S1 Fig — Double immunofluorescence on sagittal sections of gonads from XX and XY wildtype (WT) and XX Hmgcs2-null (KO (Δ647)) fetuses at 14.5 dpc for (A-C) MVH (purple, germ cells) and FOXL2 (green, pre-granulosa cells); (D-F) MVH (purple, germ cells) and SOX9 (green, Sertoli cells); and (G-I) MVH (purple, germ cells) and SYCP3 (green, meiotic germ cells). Scale bar, 100 μm. All images of fetal gonad sections are oriented so that the anterior pole is at the top and the mesonephros is on the left of the gonad. (PDF) [file pone.0227411.s001.pdf]
