# Supplementary material for: The gene encoding the ketogenic enzyme HMGCS2 displays a unique expression during gonad development in mice
Source: PLoS One. 2020 Jan 7;15(1):e0227411. doi: 10.1371/journal.pone.0227411 (PMC6946174; doi:10.1371/journal.pone.0227411)
Supplement: S1 Table — Sequences of primers used in various experiments. (DOCX) [file pone.0227411.s002.docx]

**S1 Table. Primers used in this study.** Sequences of primers used in various experiments.

| **Gene** | **Experiment** | **Forward (5’ 🡪 3’)** | **Reverse (5’ 🡪 3’)** |
| --- | --- | --- | --- |
| *Hmgcs2* | ISH probe | AAACTTCGCTCACACCTGCT | GGGCAATGTCACCACAGACCAC |
| *Hmgcs2* | qRT-PCR | CAGGAAACTTCGCTCACACC | GGAGCAGGAGGGATTGTAGA |
| *HMGCS2,* exon 1 | qPCR | GGTTTCTGCTTGCTCCTCTG | AGGTGTGAGGGAGGTTTCCT |
| *HMGCS2,* start exon 2 | qPCR | TTTCTACAGCCTCTGCTGTCC | CACATATTGGGCTGGGAAGT |
| *HMGCS2*, mid exon 2 | qPCR | AGGCTGGAAGTAGGCACTGA | TGCCTGAATCCTGGAAGAGT |
| *HMGCS2*, end exon 2 | qPCR | TGAGGGCATAGATACCACCAA | CCCAGGAACTGGACTCCATC |
| *HMGCS2*, exon 5 | qPCR | CCTTTTGCAAGATGGTCCAG | GCTGGTTTGTGTGTCACTGC |
| *HMGCS2*, start exon 10 | qPCR | GCTTCTCCCCGTGAATCATA | GCTGATGCTTATGGGGCTAC |
| *HMGCS2*, end exon 10 | qPCR | CAGCCCACTTCCCTGTTCTA | GGCAAGGAATGACCAAGAAG |
| *Fgfr2c* | genotyping | GAGTACCATGCTGACTGCATGC | GGAGAGGCATCTCTGTTTCAAGACC |
| *Hmgcs2*  Δ647 *allele* | genotyping | GGTAACTGTCAAAAGACAATGAG | CCTTTCCTAAGTTGCTCTTGC |
| *Hmgcs2*  Δ647 *control allele* | genotyping | GGTAACTGTCAAAAGACAATGAG | CCATACTTCTGTGACATCCTG |
| *Hmgcs2*  *+84 allele* | genotyping | GCAAGTTAAAATAAGGCTAGTCC | CCATACTTCTGTGACATCCTG |
| *Sexing* | genotyping | GATGATTTGAGTGGAAATGTGAGGTA | CTTATGTTTATAGGCATGCACCATGTA |
